# Genetic Gains for Grain Yield in CIMMYT’s Semi-Arid Wheat Yield Trials Grown in Suboptimal Environments

**DOI:** 10.2135/cropsci2018.01.0017

**Published:** 2018-07-12

**Authors:** L. A. Crespo-Herrera, J. Crossa, J. Huerta-Espino, M. Vargas, S. Mondal, G. Velu, T. S. Payne, H. Braun, R. P. Singh

**Affiliations:** 1 Centro Internacional de Mejoramiento de Maíz y Trigo (CIMMYT), Global Wheat Program, Apdo. 0660, Mexico City, Mexico; 2 INIFAP, Campo Experimental Valle de Mexico, Apdo. Postal 10, Chapingo, Texcoco 56230, Mexico; 3 Univ. Autonoma Chapingo, Carretera México-Texcoco Km. 38.5, Chapingo, Texcoco 56230, Mexico

## Abstract

Wheat (Triticum aestivum L.) is a major staple food crop grown worldwide on >220 million ha. Climate change is regarded to have severe effect on wheat yields, and unpredictable drought stress is one of the most important factors. Breeding can significantly contribute to the mitigation of climate change effects on production by developing drought-tolerant wheat germplasm. The objective of our study was to determine the annual genetic gain for grain yield (GY) of the internationally distributed Semi-Arid Wheat Yield Trials, grown during 2002–2003 to 2013–2014 and developed by the Bread Wheat Breeding program at the CIMMYT. We analyzed data from 740 locations across 66 countries, which were classified in low-yielding (LYE) and medium-yielding (MYE) environments according to a cluster analysis. The rate of GY increase (GYC) was estimated relative to four drought-tolerant wheat lines used as constant checks. Our results estimate that the rate of GYC in LYE was 1.8% (38.13 kg ha^−1^ yr^−1^), whereas in MYE, it was 1.41% (57.71 kg ha^−1^ yr^−1^). The increase in GYC across environments was 1.6% (48.06 kg ha^−1^ yr^−1^). The pedigrees of the highest yielding lines through the coefficient of parentage analysis indicated the utilization of three primary sources—‘Pastor’, ‘Baviacora 92’, and synthetic hexaploid derivatives—to develop drought-tolerant, high and stably performing wheat lines. We conclude that CIMMYT’s wheat breeding program continues to deliver adapted germplasm for suboptimal conditions of diverse wheat growing regions worldwide.

Wheat (*Triticum aestivum* L.) is a major staple food crop worldwide. In 2014, it was harvested on >220 million ha and produced >729 Tg of grain (FAO, 2017). The importance of wheat in the human diet is fundamental, as it provides 20% of energy and protein intake globally (FAO, 2017). However, food demand by 2050, driven by population growth and dietary changes, is projected to be 60% higher than in 2005 (Alexan-dratos and Bruinsma, 2012). Hence, meeting the global wheat demand in 2050 requires a substantial increase of grain yield (GY), which is currently ~3.3 t ha−1 (FAO, 2017). Achieving the target requires the generation of knowledge and transfer of new technologies, since expanding the cultivated land to increase production is not an option due to the adverse effects on the environment (Tilman et al., 2011). Furthermore, wheat production is constrained by the emergence of new diseases and pests, and by abiotic factors such as high temperatures and drought events, all of which are a consequence of climate change and have negative impacts on GY.

Drought is one of the most severe factors that reduce wheat productivity. It is defined as a meteorological term to denote the lack of rain for certain period, during which plants suffer from the lack of water in the soil (Munné-Bosch and Alegre, 2004). Yield loss of 20% can be observed if plants are grown with 40% less water than required to avoid the stress (Daryanto et al., 2016). However, this reduction can vary depending on the phenological stage at which the water deficit occurs; for instance, it can be larger if water is limited at reproductive stages than if limiting occurs only at the vegetative stage (Daryanto et al., 2016). Plants can experience drought when water is limited for the roots and when the transpiration rate becomes higher. At the morphological level, drought can affect germination and plant establishment, growth, biomass accumulation, leaf senescence, and GY, but at the cellular level, it affects membrane integrity, pigment content, osmotic adjustment, photosynthetic activity, gas exchange, and cell elongation (Anjum et al., 2011). Plants can cope with drought stress in various ways: (i) through an extensive root system to explore higher amounts of soil volume (Anjum et al., 2011), (ii) through osmotic adjustment and the accumulation of solutes such as proline that may improve water uptake (Keyvan, 2010; Anjum et al., 2011), (iii) by maintaining the balance between antioxidants and reactive oxygen species (ROS) levels, which can help to reduce the extent of the oxidative damage due to ROS (Anjum et al., 2011), and (iv) through induced leaf senescence as a survival mechanism to avoid drought and maintain favorable water status, which also contributes to nutrient remobilization, allowing the rest of the plant to take advantage of the accumulated nutrients of the senescent leaves (Munné-Bosch and Alegre, 2004).

Wheat improvement can significantly contribute to food security, because of the possibility of, not only increasing wheat GY potential, but also of incorporating traits (resistance and tolerance to stresses) that can protect GY and thus adequately respond to a changing climate. The Bread Wheat Breeding Program of the CIMMYT has the objective to breed for 60 million ha in developing countries (i.e., over one-quarter of the global wheat harvested area). To achieve this goal, the concept of the “mega-environment” has been crucial in classifying wheat growing environments according to common environmental features, agronomic practices, and end-use products (Rajaram et al., 1993; White et al., 2001). The drought-prone zones account for 15 million ha, classified as Mega-environment 4, considered as suboptimal conditions for wheat production. Multilocation testing and the evaluation of elite germplasm in the form of internationally grown nurseries have been fundamental to target these regions (Singh et al., 2007; Braun et al., 2010). Among other nurseries, the international Semi-Arid Wheat Yield Trial (SAWYT) is distributed annually to collaborators within the International Wheat Improvement Network (IWIN, http://www.cimmyt.org/international-wheat-improvement-network-iwin/). The SAWYTs are grown at 120 to 150 locations annually where water stress is a constraint to wheat production, with the only request of sharing data with the CIMMYT. However, frequently only 50% of data is recovered to conduct multilocation analyses.

The IWIN platform allows yearly evaluation of elite germplasm at several locations in various countries, and by including long-term checks in such trials, it is possible to evaluate the rate of GY increase relative to those long-term checks throughout a determined period (Trethowan et al., 2002; Manès et al., 2012; Sharma et al., 2012; Crespo-Herrera et al., 2017). In this way, the wheat breeding program at CIMMYT can periodically evaluate the GY of the distributed materials and the breeding progress in target environments. One consideration in the analyses of these trials is the heterogeneity of the evaluations. The IWIN collaborators follow local agronomic practices and usually do not spray trials with fungicide to control diseases, since the distributed lines are potential cultivars in those environments and are required to be tested under the prevailing conditions of each site so they can compete with currently grown cultivars. However, this can also be an indicator that superior lines across multiple locations possess yield stability, which is a key selection trait by breeding programs to develop successful cultivars. Yield stability is one among various features of CIMMYT germplasm achieved through the strategy of shuttle breeding, which aids the selection of photoperiod-insensitive and disease-resistant germplasm, and has the possibility to simulate various growing conditions at the main yield-testing site in Ciudad Obregon, Sonora, Mexico (Braun et al., 2010).

The use of mixed models for the analysis of multilocation trials aids to fit various covariance structures to the genotype × environment (GE) interaction matrix. One way of modeling GE is the factor analytical (FA) with heterogeneity of variances that uses the leading principal components of the variance-covariance GE matrix and accounts for the maximum amount of variation with a reduced number of parameters, yielding a more parsimonious variance-covariance structure (Thompson et al., 2003; Kirkpatrick and Meyer, 2004; Burgueño et al., 2008; Meyer, 2009).

The genetic relationship between individuals can be measured by the coefficient of parentage (COP), which indicates the probability that a randomly drawn allele from individual *i* is identical by descent to a randomly drawn allele form individual *j*. The COP can be estimated from the pedigree of individuals, and it contributes to the covariance between relatives if alleles are identical by descent. Covariance between relatives is fundamental since progress in selection is proportional to the resemblance between selected individuals and their progeny. The COP can also be used to determine the structure of a population with the implementation of principal component analysis (PCA), which is able to model the differences in coancestry between individuals (Price et al., 2006). However, this is more common with the use of molecular markers and the construction of the kinship matrix for genome-wide association studies.

The first objective of our study was to determine the rate of genetic gain for GY per se in 12 yr of Semi-Arid Wheat Yield Trials grown under suboptimal conditions, with the aid of mixed models and fitting a FA structure to the variance-covariance matrix of the GE. The main reason of using FA for assessing GE is that it produces estimate of means with lower SE and is thus more precise than not modeling GE (Crossa et al., 2004). The second objective was to identify the pedigree origin and ancestors of the best performing lines throughout this evaluation period by determining the COP.

## MATERIALS AND METHODS

### Data

The SAWYT is distributed annually and internationally by the CIMMYT to collaborators within IWIN. The target environments for the SAWYT selected lines are those where spring wheat is grown under suboptimal conditions with reduced water availability in rainfed or partially irrigated areas.

The analyzed trials belonged to 10th through 21st SAWYTs distributed and grown between years 2002–2003 and 2013–2014. Data were returned from 740 locations in 66 countries. Each SAWYT consists of 50 entries, of which two or three genotypes are CIMMYT checks (Table 1) plus a local check that is included in the trial by each collaborator. The remaining entries in the trial are new elite wheat lines. The elite lines are selected after 2 yr of testing under optimal irrigation conditions and for an additional 1 to 2 yr under drought and heat-stressed conditions at CIMMYT’s experimental station in Ciudad Obregon (27°37ʹ N, 109°93ʹ W). The trial design is an α-lattice with two replicates.

**Table 1:** Presence of checks in 10th through 21st Semi-Arid Wheat Yield Trials (SAWYTs).

GID^†^	Check	SAWYT											
		10th	11th	12th	13th	14th	15th	16th	17th	18th	19th	20th	21st
109278	Dharwar dry	X			X	X	X	X	X	X	X	X	X
2430154	Attila		X	X	X	X	X						
3855011	Vorobey		X	X				X	X		X	X	
197187	Cham 6	X			X	X	X	X	X	X			X

† GID, germplasm identification number.

Collaborators follow local management practices. The metadata of the trials indicate that 87% of the trials were fertilized, weeds were controlled chemically in 49% of the trials, 84% reported not to have weed problems, and 12% reported to have used insecticides. Only 5.8% of collaborators reported fungicide use, although 34.8% reported no foliar disease development, and 26, 18, and 4% reported slight, moderate, and severe disease development, respectively. Additionally, 45.8% of trials were reported to be irrigated, but no amount of water was specified. The average experimental plot was 4.5 m^2^.

Additionally, average minimum and maximum temperature and precipitation of each location was obtained from the NASA Prediction of Worldwide Energy Resource (POWER). The temperature was averaged over periods of 10 d after the planting date reported for each trial. A similar procedure was followed for precipitation, but instead of averaging, it was summed for each location in each 10-d period.

### Statistical Analysis

In general, the sequence of analyses and the statistical models used to measure realized genetic gains for the SAWYT datasets are similar to those used by Crespo-Herrera et al. (2017). In this section, we briefly explain the models and methods.

The GY data of the trials was analyzed with the R software (R Development Core Team, 2013) with the ASREML-R package (Butler et al., 2009).

**Fig. 1:**
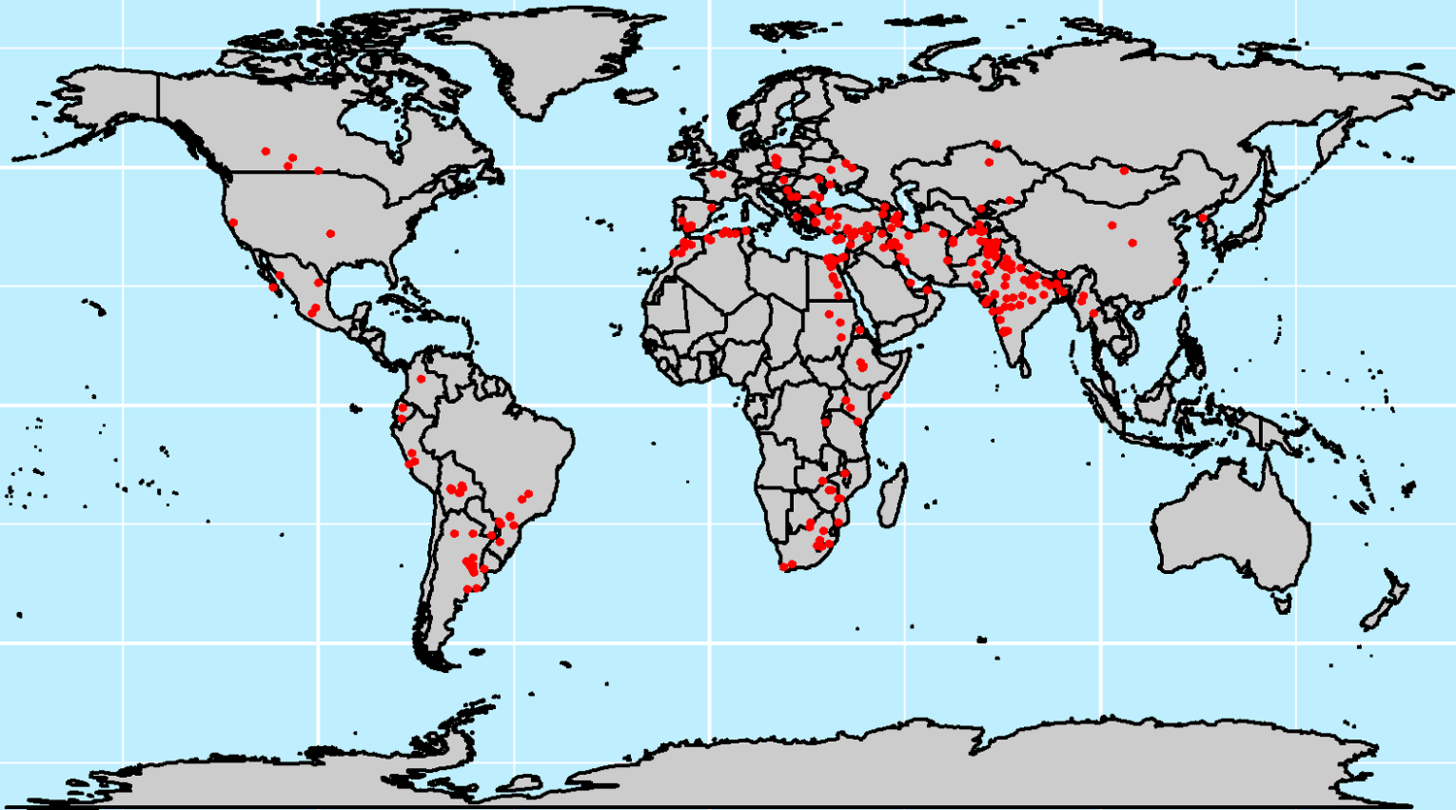
Locations where Semi-Arid Wheat Yield Trials (SAWYTs) were grown from 2002–2003 (10th SAWYT) to 2013–2014 (21st SAWYT).

The trials were first analyzed individually:

$$Y_{ijk}=\mu +R_{j}+SB_{k}(R_{j})+G_{i}+\varepsilon_{ijk}$$ [1]

where µ is the general mean, G_i_ is the fixed effects of the genotypes (*i* = 1, ..., 50), Rj is the fixed effects of the replicates (j = 1, 2), SB*_k_* represents the random effects of the sub-blocks (*k* = 1, ..., 5), assumed to be independently and identically normal distributed with mean zero and variance σ^2^*_sb(r)_*. The term ε*_ijk_* is a random residual assumed to be identically normal distributed with mean zero and variance σ^2^_ε_. Repeatability was estimated from the variance components of the individual site analyses to discard all locations with repeatability <0.05 from further analyses. This left a total of 486 trials in 45 countries (Fig. 1).

After the individual sites were analyzed, the mean GY of each trial was used in a k-means clustering analysis (k = 3) to determine groups of trials grown in high-, medium-(MYE), and low-yielding environments (LYE). Environments classified as high yielding were not used in the analyses because of the reduced number of locations, between 2 and 12. Hence, results are only presented for environments classified as LYE and MYE, which are the targets of SAWYT germplasm.

Once the sites within each SAWYT were grouped into LYE and MYE environment type based on yield level, they were analyzed in a combined manner (sites within environment type) and also across environment type:

$$Y=Xb+Z_{r}r+Z_{g}g+Z_{ge}ge+e$$ [2]

where **X** is the incidence matrix for the fixed effects of sites, and **Z_r_**, **Z_g_**, and **Z_ge_** are the incidence matrices of zeros and ones for the random effects of replicates within sites, genotypes, and GE, respectively. Vector **b** denotes the fixed effect of sites; vectors **r, g, ge**, and **e** contain random effects of replicates within sites, genotypes, GE, and residuals, respectively, and are assumed to be random and multivariate normal distributed with zero mean vectors and variance-covariance matrices **R, G, GE**, and **E**, respectively.

The variance-covariance matrices **R, G**, and **E** are assumed to have a simple variance-covariance component structures such that the replicates within sites are R = **Σ_r_** ⨂ **I_r_**, where **Σ_r_** = diag($\sigma_{r_{j}}^{2},j=1,2,\cdots ,s$) and **I_r_** is the identity matrix of order *r*, the residual variance is $E=\sigma_{e}^{2}I$, where $\sigma_{e}^{2}$ is the independent and identical error variance, and the line variance is $G=\sigma_{g}^{2}I$, where $\sigma_{g}^{2}$ is the genetic variance assuming no relationship between lines.

The GE interaction variance is modeled as **GE** = **𝚺_ge_** ⨂ **I** interaction matrix variance-covariance, which can be represented as

$$\begin{array}{l} GE+\sum_{ge}⊗I=\\ \left[\begin{array}{llll} \sigma_{ge_{1}}^{2} & \rho_{12}\sigma_{ge_{1}}\sigma_{ge_{2}} & \cdots & \rho_{1s}\sigma_{ge_{1}}\sigma_{ge_{s}}\\ \rho_{12}\sigma_{ge_{1}}\sigma_{ge_{2}} & \sigma_{ge_{2}}^{2} & \cdots & \cdot \\ \cdot & \cdot & \cdots & \cdot \\ \cdot & \cdot & \cdots & \cdot \\ \cdot & \cdot & \cdots & \cdot \\ \rho_{s1}\sigma_{ge_{s}}\sigma_{ge_{1}} & \cdot & \cdots & \sigma_{ge_{s}}^{2} \end{array}\right]⊗I \end{array}$$

where the *j*th diagonal element of the *s* × *s* matrix **𝚺_ge_** is the GE variance $\sigma_{ge_{j}}^{2}$ in the *j*th site, and the *j*th element is the GE variance covariance ρ*_ij_*𝜎_g_*_e_i__* 𝜎*_ge_j__* between Sites i and *j*; thus, ρ*_ij_* is the correlation of GE variance effects between Sites i and *j*. The matrix **I** is of order g × g assuming that the lines are not related and the breeding value of each genotype will be predicted only by the value of the empirical response of the genotype itself.

### Factor Analytic Model

The interaction matrix **GE** can be modeled using different structure matrices; the most liberal structure is the completely unstructured model, which assumes that matrix **GE** contains *s*(*s* – 1)/2 parameters, whereas the most restrictive variance-covariance structure is to assume that **GE** variances within all sites are equal, and all pairwise correlations between genotypes and between sites are zero. A parsimonious variance-covariance structure is the FA structure that models covariance among observations in terms of a few hypothetical unobserved factors and is useful for modeling **GE**. The FA model has been extensively used for modeling **GE** (Smith et al., 2002; Crossa et al., 2004).

The FA structure with k ≤ *s* factors (where s refers to the *s*th site) or components [FA(*k*)] takes the form **△△ʹ** + **D**, where **△** is an *s* × *k* matrix of δ*s*, and D is an *s* × *s* diagonal matrix with *s* possibly unique and different nonnegative parameters on the diagonal. Each column of **△** contains the genotype scores for one of the multiplicative terms. For *k* = 1, the model FA(1), has one multiplicative term; for *k* = 2, model FA(2); and so on for FA(3), etc. (Crossa et al., 2006; Burgueño et al., 2008). Boundary constraints must be imposed on the solutions to obtain a unique solution. This is achieved by fixing some estimates as having values of zero. A full description of FA can be easily found in several publications.

### Expressing the Line Best Linear Unbiased Predictor as a Percentage of the Checks

The best linear unbiased predictors (BLUPs) were obtained from the analysis and then expressed relative to the GY of checks:

$$GYC=\left(\frac{BLUP-MeanGY}{MeanGY}\right)\times 100$$ [3]

where BLUP represents the predicted GY value of a wheat line, and Mean GY represents the predicted GY mean of the checks. After calculating the GYC, the 10% of the highest yielding lines (HYLs) were selected to regress the GYC on the years of SAWYT distribution to estimate the annual GYC increase rate in each environment (LYE and MYE), and also across the locations of these environments.

### Genetic Relationships

The COP of the HYLs was estimated as described by Cockerham (1983) with the Browse application of the International Crop Information System (ICIS) software described in McLaren et al. (2005). A PCA was performed with the square matrix of the COP to identify groups of genotypes that are more related among themselves and to determine which groups of genotypes is more frequent among the HYLs throughout the analyzed period. This will give information on the most predominant crosses included in the historical set of SAWYTs.

## RESULTS AND DISCUSSION

### Semi-Arid Wheat Yield Trial Distribution

Between 2002–2003 and 2013–2014, there were 428 SAWYTs analyzed (Table 2), of which 216 (50.5%) were classified to be under LYE and 212 (49.5%) under MYE. High-yielding environments (58) were not considered in the analysis. According to the meteorological data obtained from POWER, the LYE and MYE patterns of temperature and precipitation were similar (Supplemental Fig. S1 and S2, which show that at 90 d after planting, the minimum temperature remained below 11.0°C and precipitation was <200 mm throughout a period of 120 d).

**Table 2:** Number of locations per yield environment in the 10th through 21st Semi-Arid Wheat Yield Trials (SAWYTs).

Yield environment	SAWYT												Total
	10th	11th	12th	13th	14th	15th	16th	17th	18th	19th	20th	21st	
Low	15	17	17	17	14	21	15	18	22	16	24	20	216
Medium	13	10	15	9	14	7	17	27	21	25	30	24	212
Total	31	32	34	29	30	32	36	51	55	45	61	50	428

**Fig. 2:**
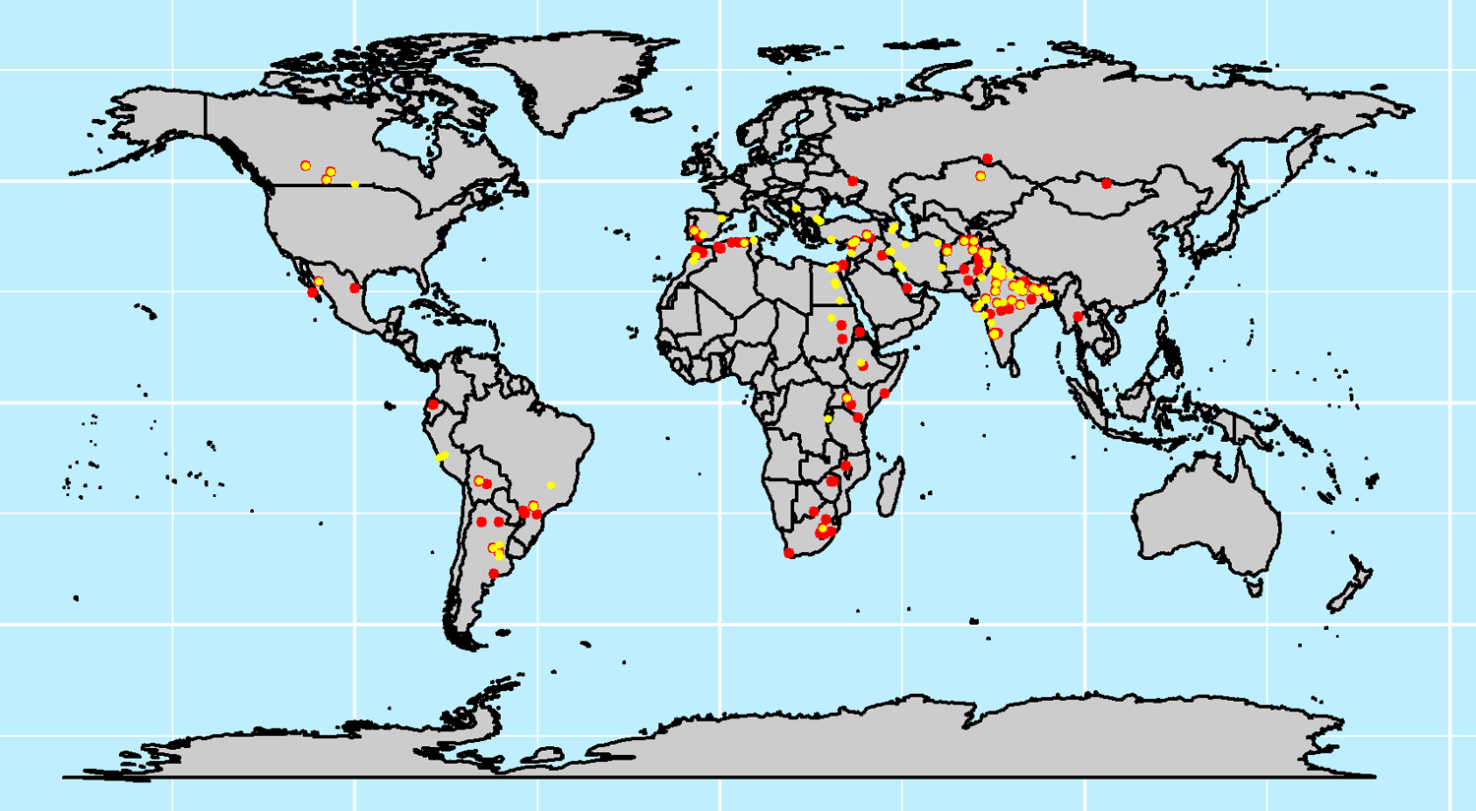
Location of the two yielding environments where Semi-Arid Wheat Yield Trials (SAWYTs) were grown from 2002–2003 (10th SAWYT) to 2013–2014 (21st SAWYT). Red and yellow points indicate low- and medium-yielding environments, respectively.

**Fig. 3:**
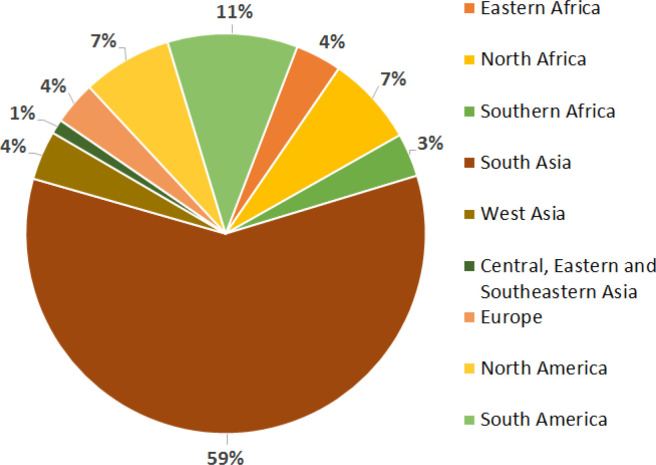
Global distribution of 428 Semi-Arid Wheat Yield Trials (SAWYTs) grown from 2002–2003 (10th SAWYT) to 2013–2014 (21st SAWYT).

Trials were grown in 45 countries (Fig. 2), and 59% of them were located in South Asia, followed by South America (11%) and then North Africa and North America (7%) (Fig. 3). In South Asia, 42.7% were classified as LYE, and 57.3% were classified as MYE. In South America, 57.8 and 42.2% were LYE and MYE, respectively. In North Africa, 61.3% were LYE and 38.7% were MYE. The proportion in North America for each respective environment was 51.3 (LYE) and 48.4% (MYE). Although, these trials are designed for suboptimal environments, it is often requested by multiple partners to grow them in optimal environments, hence the need to classify based on the mega-environment and the actual GY. These regions represent important wheat production environments for CIMMYT’s Wheat Breeding Program, given that South Asia accounts for ~23% of the global harvest area, 13% is harvested in North America, and 3% each is harvested in North Africa and South America (FAO, 2017). Furthermore, one of the effects of an increased global temperature is the higher risk of inducing drought and/or reduced rainfall events in South Asia, particularly in Pakistan, northwestern India, and Afghanistan (Akbari et al., 2006; Annamalai et al., 2013), hence the importance of continuous wheat breeding, yield testing, cultivar release, and technology transfer as some of the measures to mitigate the impacts of climate change on wheat productivity, since they can be devastating particularly in South Asia and North Africa, where wheat yield could be reduced by 17% (Knox et al., 2012).

In addition to extreme climatic events, diseases and pests can also significantly reduce wheat productivity. From the metadata of the trials, we found that 4% of all trials were treated with fungicide in LYE, whereas 1.9% were treated in MYE. Additionally, 19.4 and 15.4% of all trials reported not to have foliar disease problems in LYE and MYE, respectively. Only 2.6 and 1.9% had severe foliar disease infections in LYE and MYE, respectively. This indicates that, in general, trials were not highly affected by leaf diseases, which can result from the fact that disease resistance is paramount for the wheat breeding program at CIMMYT. However, numeric data are required to fully account for this aspect in the analysis, which are not regularly returned by the cooperators.

### Grain Yield and Genetic Gains

**Fig. 4:**
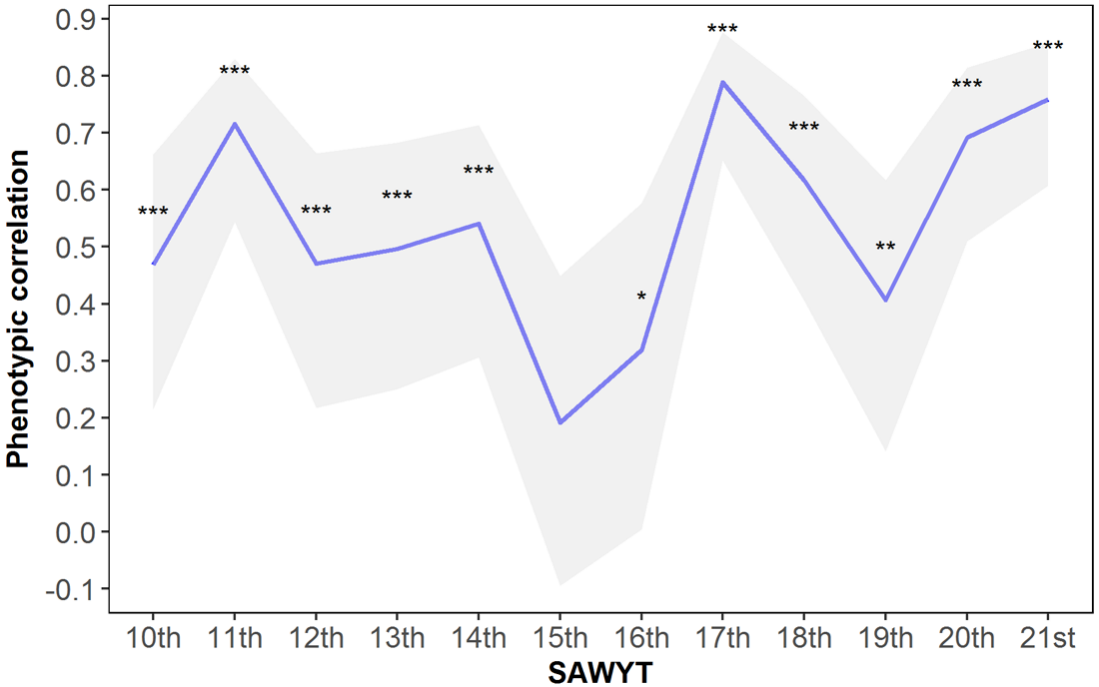
Phenotypic correlations between low- and medium-yielding environments for each Semi-Arid Wheat Yield Trials (SAWYTs) grown from 2002–2003 (10th SAWYT) to 2013–2014 (21st SAWYT). 95% confidence intervals are indicated by the shaded area. Significance levels: *** *p* < 0.001, ** *p* < 0.001, * *p* < 0.05.

The average GY of the trials ranged from 0.15 to 3.5 t ha^−1^ for the LYE, whereas the average yield for the MYE ranged from 3.53 to 6.0 t ha^−1^. The cluster centers were 2.4 and 4.6 t ha^−1^ for LYE and MYE, respectively. The average GY of the checks ranged from 1.7 to 2.6 t ha^−1^ and 3.8 to 5.3 t ha^−1^ in LYE and MYE, respectively. The average GY of the checks across environments ranged between 2.8 and 3.6 t ha^−1^. Phenotypic correlations between both yielding environments were in all cases positive and significant (*p* < 0.05), except for the GY data for the 15th SAWYT (Fig. 4). This reinforces the fact that it is possible to simultaneously breed for optimal and suboptimal conditions (Singh et al., 2007; Singh and Trethowan, 2007), so the lines will be able to perform well under the uncertainties of the year-to-year weather.

The multilocation analysis of the SAWYTs is paramount for making breeding decisions (selection, crossing, yield testing, etc.,), and because it is annually distributed, SAWYTs are also useful to periodically measure the rate of genetic gain for GY per se that has been achieved over certain period. Unlike previous international trial evaluations (Manès et al., 2012; Sharma et al., 2012), in our present study, we conducted the GY data analyses in a different period considering more recently distributed SAWYTs to determine the progress of recently distributed germplasm. We also estimated the rate of GY increase considering the performance of various checks included in the trials. Additionally, to gain precision in the estimation of BLUPs and reduce the dimensionality of GE, we fit a FA structure to the GE covariance matrix and used cluster analysis for the classification of environments based on their GY level.

**Fig. 5:**
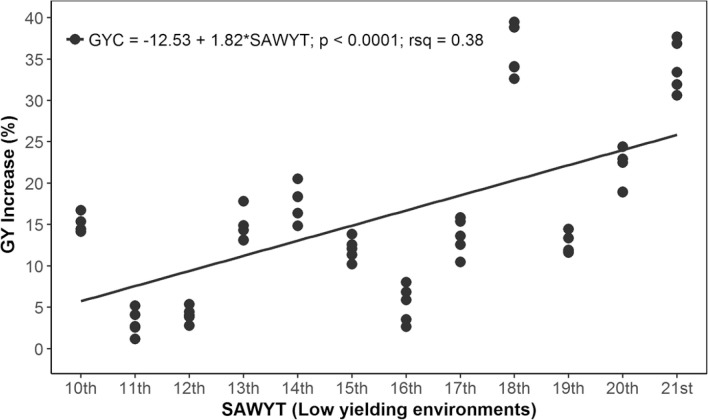
Grain yield (GY) increase of the highest yielding lines selected for low-yielding environments throughout 2002–2003 (10th Semi-Arid Wheat Yield Trial [SAWYT]) to 2013–2014 (21st SAWYT). GYC is the GY relative to checks; rsq is *R*_2_.

**Fig. 6:**
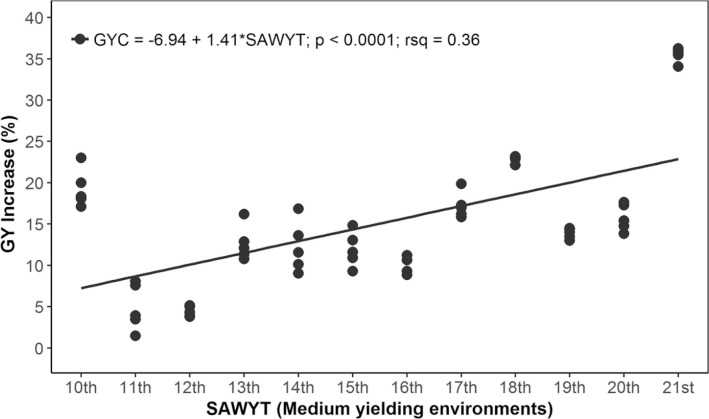
Grain yield (GY) increase of the highest yielding lines selected for medium-yielding environments throughout 2002–2003 (10th Semi-Arid Wheat Yield Trial [SAWYT]) to 2013–2014 (21st SAWYT). GYC is the GY relative to checks; rsq is *R*_2_.

**Fig. 7:**
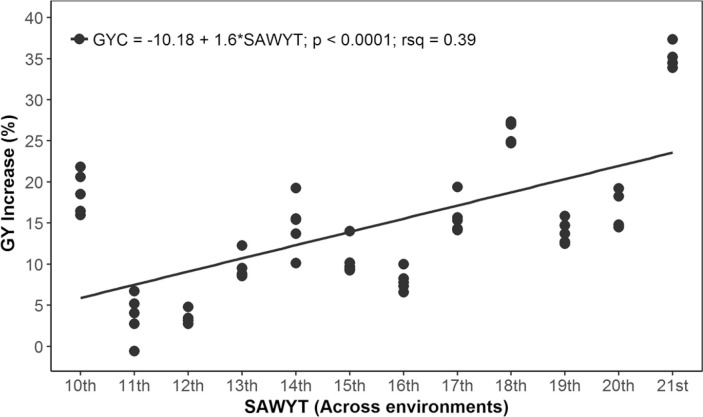
Grain yield (GY) increase of the highest yielding lines selected across environments throughout 2002–2003 (10th Semi-Arid Wheat Yield Trial [SAWYT]) to 2013–2014 (21st SAWYT). GYC is the GY relative to checks; rsq is *R*_2_.

Our results indicate that the rate of GYC increase in LYE was 1.8% annually (Fig. 5), that is, 38.13 kg ha^−1^ yr^−1^. The increase of GYC in MYE was 1.41% per annum (Fig. 6), or 57.71 kg ha^−1^ yr^−1^. The increase of GYC Across environments was 1.6% (Fig. 7), or 48.06 kg ha^−1^ yr^−1^. In all cases, the slope of the regression was highly significant (*p* < 0.0001), and the *R*^2^ was between 0.36 and 0.39. The evaluation of SAWYT made by Manès et al. (2012) between 1994 and 2010 showed a rate of genetic GY increase across sites was between 1 and 1.5%; in LYE, it was 0.5 to 1.1%, and in high-yielding environments, it was 1.2 to 1.7%, taking as a reference the drought-tolerant checks ‘Dharwar Dry’ and ‘Cham 6’. Cultivar Dharwar Dry from India is reported to be drought tolerant (Kirigwi et al., 2007). The wheat cultivar Cham 6 was released in Syria in 1991 and also in Jordan with the name of ‘Nesser’, and it was recommended for cultivation in rainfed areas (Bishaw et al., 2011). However, as wheat breeding moves forward, in later SAWYTs (11th and 12th), a new line (‘Vorobey’) was included in the evaluations, which ranked top among these trials and showed a high performance in ~70% of all evaluated sites (Manès et al., 2012). Thus, it was also included as check in some later trials (Table 1). The additional check in our study was cultivar ‘Attila’ from 11th to 15th SAWYT (Table 1), still a widely grown cultivar (Lantican et al., 2016) characterized by its yield stability in various environments, but now moderately susceptible to yellow rust (Puccinia striiformis Westend.) races virulent to the Yr27 gene (Singh et al., 2012).

Various methodologies exist to measure the rate of genetic gains, all of which are restricted to the dimension of economic resources that can be allocated to conduct evaluations. A common method is the selection of elite germplasm or released cultivars at different time points over a large span, planted in an appropriate experimental design for various crop seasons, under fungicide and insecticide treatments and without water and fertilizer limitations, to avoid the masking effect of resistance to diseases and tolerance to other stresses (Fischer et al., 2014). This methodology has been used by various authors, and the reported rate of genetic GY gains in spring wheat ranged from 0.59 (30 kg ha^−1^) to 0.9% (64 kg ha^−1^) per year (Sayre et al., 1997; Lopes et al., 2012; Aisawi et al., 2015). Other reports on winter wheat indicate gain rates between 0.9 and 1.4% (14.6-46.7 kg ha^−1^) relative to cultivar ‘Kharkof’ in the United States (Green et al., 2012; Battenfield et al., 2013), and up to 1.2% (72.1 kg ha^−1^) in China (Zhou et al., 2007). Another possible methodology is the evaluation of long-term national trials, which are frequently conducted in different countries with the main objective of cultivar release, reporting, for instance, yield gains from 0.13% (36 kg ha^−1^) in Finland to 0.9% (71 kg ha^−1^) in Germany (Peltonen-Sainio et al., 2009; Laidig et al., 2014; Piepho et al., 2014).

### Pedigree Relationships

There were 102 advanced lines that were the HYLs throughout all SAWYTs, of which 48 (47%) ranked top in both environments and/or in one environment and across all trial sites. Full pedigrees, GY, GYC, and ranks by environment of all HYLs are presented in the Supplemental Table S1.

**Fig. 8:**
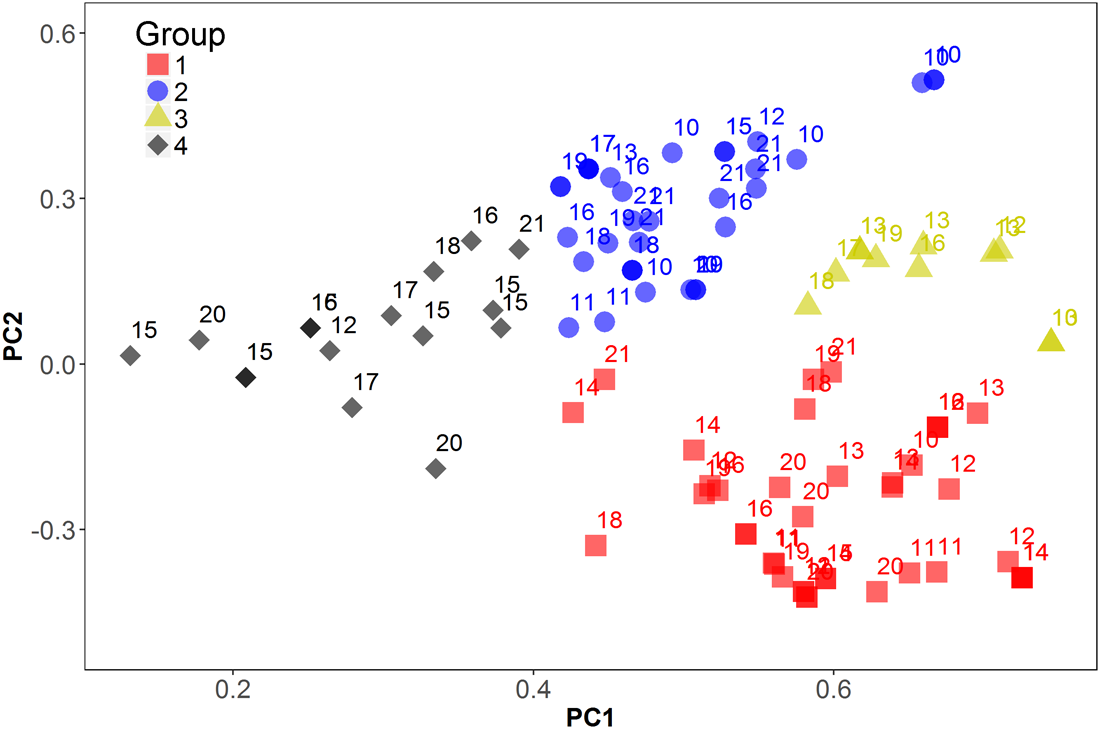
Principal components analysis plot for the coefficient of parentage of the highest yielding lines in all analyzed Semi-Arid Wheat Yield Trials (SAWTs). Groups indicate the germplasm common ascendant lines (1 = Pastor; 2 = Babax; 3 = Pastor/Babax; 4 = synthetic hexaploids wheat). Numbers above the symbols indicate the SAWYT number to which genotypes belong. PC1 and PC2 are the principal components.

The PCA results of the COP with the HYLs throughout all analyzed SAWYTs indicated the separation of the lines in four groups (Fig. 8). After examining the pedigrees of the lines, these groups enclosed germplasm derived (i) from a CIMMYT line named ‘Pastor’ and related progenitors (i.e., progenitors of Pastor), (ii) from a Mexican cultivar of CIMMYT origin named ‘Baviacora 92’ (Babax) and related progenies (i.e., progenies of Baviacora 92), (iii) from both ancestors, Pastor and Baviacora 92, and (iv) from progenitors that were crossed with synthetic hexaploid wheat-derived lines. Of the total 102 HYLs, 39.2% (40 lines) were clustered in Group 1, 34.3% (35 lines) in Group 2, 10.8% (11 lines) in Group 3, and 15.7% (16 lines) in Group 4 (Table 3). Wheat lines derived from Pastor and Baviacora 92 have been identified for their drought tolerance in other studies, and their derivatives have been used extensively in breeding for their yield stability (Singh et al., 2007; Olivares-Villegas et al., 2007; Manès et al., 2012). In fact, quantitative trait loci for yield-related traits under heat and drought stress have been identified in the background of Babax (Pinto et al., 2010). Synthetic hexaploid wheats have been reported to improve drought tolerance by conferring adaptive traits such as increased root mass at depth, water use efficiency, and water extraction capacity (Lopes and Reynolds, 2011). Our results indicate that wheat breeding at CIMMYT has used previously known sources of drought tolerance and also incorporated the diversity originating from wheat wild relatives through the exploitation of synthetic hexaploid wheat-derived lines, thus implementing a strategy in which drought adapted germplasm is developed based on a wider genetic base.

**Table 3:** Number of highest yielding lines by yielding environment (low- [LYE] or medium-yielding [MYE]) clustered in four principal component analysis groups.

Group	LYE	MYE	Across	Total per group
1	25	26	27	40 (39.2%)
2	18	21	17	35 (34.31%)
3	6	4	7	11 (10.8%)
4	8	7	7	16 (15.7%)
Total	57	58	58	102 (100%)

In conclusion, CIMMYT’s bread wheat breeding program continues to develop and deliver adapted germplasm for suboptimal conditions through the distribution of SAWYTs. Our results are in the range of other observations that have measured genetic gains of GY in different countries and environments. The progress of GY under rainfed conditions is being achieved by the utilization of well-known drought-tolerant and yield-stable germplasm, as well as through the exploitation of the genetic diversity originating from wheat wild relatives in the form of synthetic hexaploid wheats.

## Abbreviations

BLUP: best linear unbiased predictor
COP: coefficient of parentage
FA: factor analytical
GE: genotype × environment
GY: grain yield
GYC: grain yield relative to checks
HYL: highest yielding
IWIN: International Wheat Improvement Network
LYE: low-yielding environments
MYE: medium-yielding environments
PCA: principal component analysis
POWER: Prediction of Worldwide Energy Resource
ROS: reactive oxygen species
SAWYT: Semi-Arid Wheat Yield Trial.
