# Supplementary material for: Genetic Gains for Grain Yield in CIMMYT’s Semi-Arid Wheat Yield Trials Grown in Suboptimal Environments
Source: Crop Sci. 2018 Jul 12;58(5):1890–8. doi: 10.2135/cropsci2018.01.0017 (PMC7691759; doi:10.2135/cropsci2018.01.0017)

Figure S1. Average minimum and maximum temperatures in LYE and MYE over 10-day periods after the planting date of each single SAWYT.

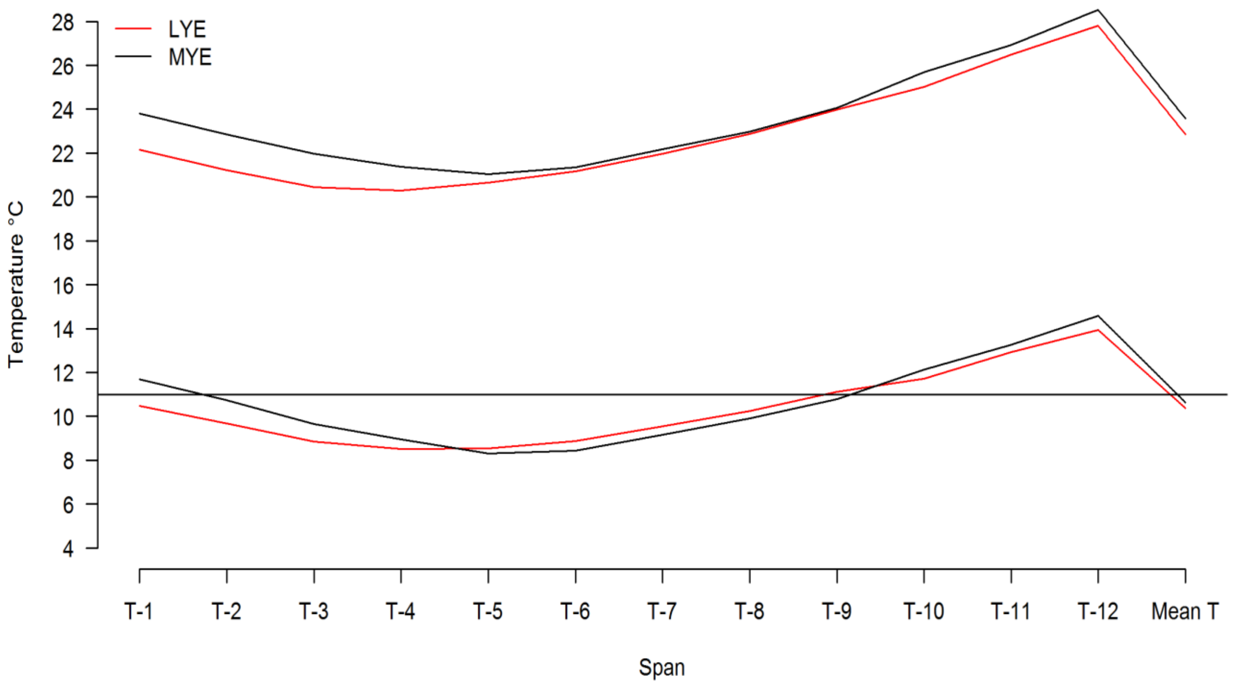

Figure S2. Average and total average precipitation in LYE and MYE over 10-day periods after the planting date of each single SAWYT.

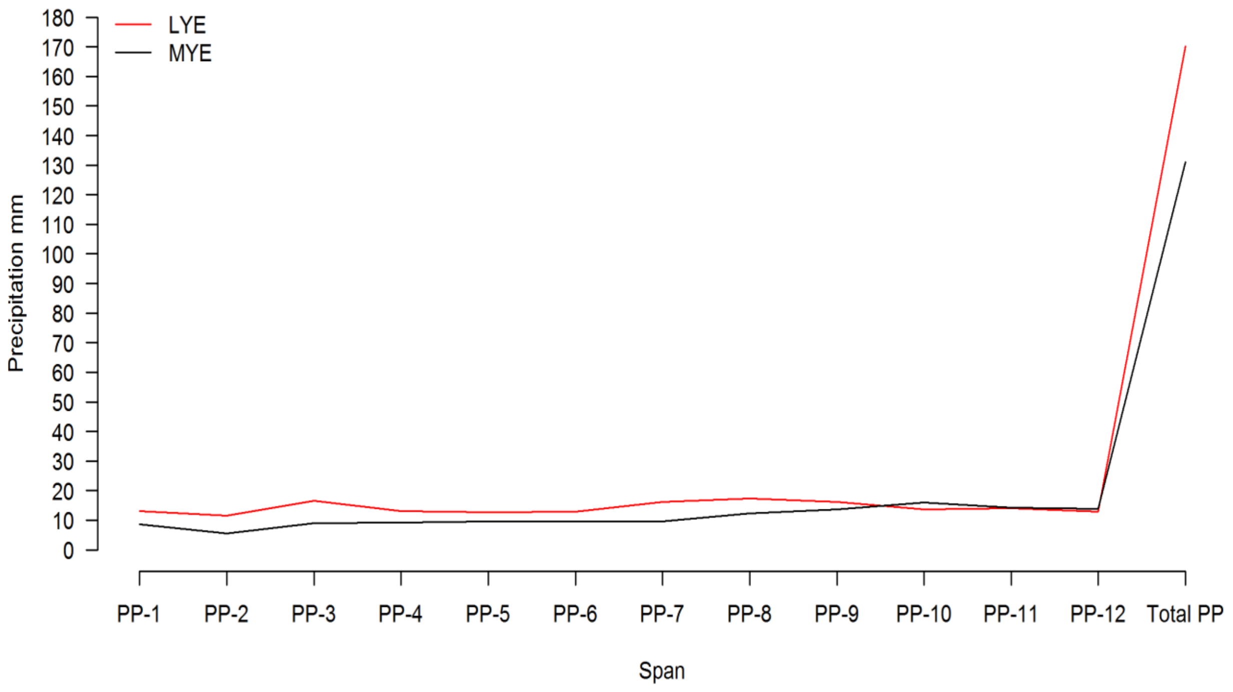

Supplement: Supplementary file 1 [file CROPSCI-58-05-1890-s001.pdf]
